# Reducing Problematic Substance Use under Exceptional Circumstances—Effects of the COVID-19 Restrictions on Inpatient Substance Use Disorder Treatment in Finland

**DOI:** 10.3390/ijerph191811436

**Published:** 2022-09-11

**Authors:** Janika Kosonen, Eeva Ekqvist, Katja Kuusisto

**Affiliations:** Faculty of Social Sciences, Tampere University, 33100 Tampere, Finland

**Keywords:** alcohol, drugs, COVID-19, desistance, substance abuse treatment, inpatient

## Abstract

The COVID-19 pandemic has affected people’s daily lives on multiple levels. At highest risk are the most vulnerable members of the society, whose lives were already affected by various risks even before the pandemic. This study investigates *how clients in inpatient substance use disorder treatment experienced the COVID-19 restrictions and their influence on recovery.* The research data consists of six focus group interviews conducted remotely using a semi-structured thematic interview method. The focus group clients (*N =* 19) were currently in inpatient substance abuse treatment during the pandemic and the ensuring restrictions. The data were analyzed using qualitative content analysis. The results show that the COVID-19 restrictions have influenced the clients’ desistance processes throughout the pandemic. The restrictions seemed to exacerbate substance abuse problems before treatment initiation and highlight the importance of peer support during treatment. Moreover, the restrictions seemed to change the function and hamper the management of social capital, raise concerns about returning home, as well as intensifying the inter-municipal segregation of services. To conclude, attention should be paid to facilitating and ensuring informal support and managing social capital. In light of this study, it seems necessary to explore the social conditions among clients in inpatient substance abuse treatment during the pandemic.

## 1. Introduction

The COVID-19 pandemic began to spread globally at the end of 2019. From the beginning of 2020, national governments initiated discussions and contemplations on arrangements to prevent the virus from spreading [1,2,3]. As in other countries, several restrictive measures were imposed by the Finnish government. These restrictions included, for example, school lockdowns, regional isolations, and restrictions on the opening hours of restaurants and bars. The restrictions due to the COVID-19 pandemic significantly affected interpersonal interaction and social life. Due to the restrictions, the virus affected not only public health but also the social, cultural, economic, and political dimensions, of human life [4,5].

In such a global crisis, the most vulnerable citizens, including people with substance use disorder (SUD) (most of the patients are referred to treatment from social services. Hence, they may not have a diagnosis of substance use disorder and the criteria for accessing the treatment vary depending on their municipality of residence), are at particularly high risk [3]. These people have faced various health-related risks, need for social support, and uncertainties in their everyday lives, even before the pandemic [6]. In addition to the fact that people experiencing SUD are at high risk for both contracting the disease and COVID-19 related mortality [2,3,7], they are also likely to experience poor social conditions and are susceptible to certain somatic and mental illnesses [8]. Poor social conditions can mean, for example, financial or housing issues, or difficulties related to social relationships, like social deprivation. Furthermore, these people may have restricted access to crucial information or adequate hygiene during the pandemic. Restricted access to information may be related to fewer opportunities to use telecommunication tools [9]. The pandemic has further accentuated these factors [4,10].

Professionals have stressed the severity of the harmful effects of the pandemic on people experiencing SUD. As the pandemic can be expected to further emphasize factors that increase risks among people with SUD, the importance of adequate treatment and services also increases [8]. However, SUD services and service providers have faced major pandemic-related challenges under exceptional circumstances, including the lack of competent staff and problems in enrolling new clients [9,11].

Entering inpatient SUD services usually indicates that outpatient services have proven insufficient, which reveals something about the complexity of the clients’ life situations [12]. Inpatient SUD services aim to offer treatment and support in a recovery-oriented environment, during which the individual undergoes an identity change process.

The identity change process has also been deemed essential in criminological desistance theories, which we use as a theoretical frame in this study. Desistance is a criminological concept that refers to the process of ceasing to commit crimes or detaching from a criminal lifestyle [13,14]. However, desistance theories have also been applied to substance use and addiction research see e.g., [15,16,17,18]. Desistance from substances refers to the process of becoming detached from a lifestyle that includes problematic substance use or simultaneous processes of desistance from crime and problematic substance use. By implementing desistance theories in the context of recovery we do not seek to promote the idea of substance use as criminal behavior. Instead, desistance theories are used because they offer an alternative approach to examining the phenomena of becoming detached or recovering from harm producing activities.

Desistance, whether understood in the context of criminal activities or substance use, represents a complex process rather than a single outcome [15,17,19,20]. The concepts of primary, secondary, and tertiary desistance, have been proposed to distinguish different procedural phases. Primary desistance refers to the phase when a person abandons substance use, while the secondary desistance refers to maintaining the change. The tertiary phase requires a successful identity change, and a new identity to become confirmed and approved. Through this process, the individual reintegrates into and engages with the community they live in [15,16,21]. The procedural nature of recovery has also been recognized in research concerning rehabilitation e.g., [21,22].

An important concept in desistance theories is social capital. Social capital is capital of social connections; it is an individual resource created in inter-personal relations and plays an important role in the development of human capital. Defining social capital is complex, because it is the least concrete form of individual resources as it is produced in human relations and interactions [23,24]. Farrall [25] has affirmed the importance of an individual’s intrinsic motivation along with alterations in their social conditions. He notes that the utilization of human capital is difficult, insofar as social and economic conditions do not encourage such a change. Social capital is also deemed one of the most critical key concepts in the recovery literature. Along with physical, human, and cultural capitals, social capital is seen as an essential element in recovery capital, which originally draws on social capital [24,26,27]. The underlying idea of recovery capital is the various resources considered supportive regarding an individual’s recovery process [27,28].

Inpatient SUD treatment aims to improve the clients’ well-being and coping skills on multiple levels [12,29,30,31]. Case management is frequently based on cognitive-behavioral methods, which support individuals in reducing substance use and discovering alternative manners in situations which used to include substance use [32,33]. In Finland, the longer-term inpatient SUD treatment usually follows a shorter detoxification treatment period that commonly lasts for one to two weeks. The longer-term inpatient SUD treatment, which is the subject of this study, is more rehabilitative in nature and draws on the sense of community and relatedness. The treatment unit personnel tend to have close contact with the clients. However, the pandemic has caused major challenges for these professionals as well. These challenges include, for example, issues in preventing the virus from spreading, arranging conditions for social distancing, and co-operating with other professionals [34], see also [8,30].

In this article we focus on the clients’ perspectives. This study aims to ascertain *how clients in inpatient SUD treatment have experienced the COVID-19 restrictions and their influence on recovery.* The framework draws on some of the criminological desistance theories applied to the context of recovery.

## 2. Participants and Data

This study was conducted in two inpatient SUD treatment units located in Finland as part of a research project *Change in client’s well-being and rehabilitation activities in inpatient substance abuse treatment.* The treatment units provide non-medical, therapeutic, community-based treatment for both individuals and families. The treatment applies cognitive behavioral therapy; the focus is on providing information about recovery, relapse, and behavioral patterns, in order to achieve change in problematic substance use. Therapeutic communities are both a way of organizing daily practices during treatment and a therapeutic method, including group sessions in addition to individual sessions with employees. Participants are also encouraged to take part in 12-step groups. Treatment periods usually last from one to three months, but for families they may be longer. Municipalities bear the majority of the treatment costs, and referral to treatment is usually from public health and social services.

The research data consists of six focus group interviews (see Table 1), which, due to the COVID-19 restrictions, were conducted remotely in the spring of 2021 via Zoom through semi-structured thematic interviews. All Zoom sessions were recorded and transcribed. The interviews were conducted by two social work researchers from Tampere University, who were working on the research project at the time. All clients (*n =* 19) were currently in inpatient SUD treatment, which was the main determinant for the selection of study participants. The participants were recruited from two Finnish inpatient SUD treatment units and all clients who wanted to participate in the study were selected.

Each focus group included three participants, except for one group that included four participants. The average duration of a single focus group interview was about 65.8 min (43–83 min) (see Table 2). The transcribed interviews amounted in total to approximately 139 pages (Times New Roman, font size 12, and spacing 1.5). Participants gave informed consent either in writing or verbally on record.

All participants took part in the study voluntarily and were informed about the research procedures both in writing and verbally before each interview session. Research permission was obtained on 15 December 2020 from the background organization of the treatment units. Participants’ consent was requested after they had been informed about the study. They were free to withdraw from the study at any stage. The research complied with the guidelines of the Finnish codes of research ethics and governance [35,36] and with the codes of research integrity in Europe [37].

## 3. Method

The data were analyzed by means of content analysis and Atlas.ti 8.4.4 was used. In analyzing the data, desistance theories are used as an analytical frame to observe factors that support and encourage recovery. The data were reread multiple times and constantly revisited.

Desistance theories framed the analysis. The various perspectives of desistance directed the formation of the thematic body of analysis. The theoretical background guided the analysis and assisted in creating an understanding of the clients’ experiences. The procedural nature of desistance was conductive to a chronological reading of the data.

First, we created three chronological codes (1) before treatment, (2) during treatment, and (3) after treatment. Then, we coded the whole data with these codes to arrange the data in chronological order. Following on, we created a series of content codes relying on the desistance theories. Eventually, five content codes were created: (1) identity change process, (2) turning points, (3) procedural support, (4) social capital, and (5) reintegration into the community. Chronological coding revealed altogether 355 citations of which 79 citations corresponded directly to the content codes above. We took all these 355 citations for closer scrutiny.

We included desistance theories in the analysis by creating the codes according to their key principles. These principles directed our attention when we analyzed the data. However, the analysis also contained inductive features as the final contents that we identified from the data were observed from clients’ subjective experiences, which they talked about in the interviews. We were able to identify the following contents: (1) applying for assistance, (2) social capital management and informal procedural support, (3) identity, (4) technology, and (5) inter-municipal segregation. These contents emerged consistently in the data.

When presenting the analysis, the participants’ quotations are distinguished by a letter (M = man, W = woman, and A = interviewer), together with a serial number, e.g., M1, W1, or A1. The interviews (I = interview) are marked with a serial number, e.g., I1. The results are presented so that one paragraph includes excerpts from one focus group interview at a time.

## 4. Clients’ Experiences of the Influence of COVID-19 and the Restrictions It Necessitated

This research is based on six focus group interviews which were conducted remotely via Zoom. There were altogether 19 participants in the study, of whom nine were women and 10 were men. The youngest participant was 23 years old, and the oldest participant was 45 years old, the average age being approximately 33.5 years (See Table 1). The participants were recruited indirectly by the treatment unit personnel.

In this research, we are primarily interested in the clients’ subjective experiences of the COVID-19 restrictions and their effects. Five themes stood out. Firstly, the restrictions seem to influence the participants’ life considerably before they entered treatment. In some cases, these major impacts caused the individual to seek help. Secondly, the restrictions influenced social capital management and supply for informal procedural support. Third, the analysis revealed the participants’ prevailing identity change processes. The fourth theme that emerged from the data was the increasing role of technology and its influence on interaction. Eventually, the participants seem to be affected by intensified inter-municipal segregation due to the restrictions.

### 4.1. Applying for Assistance

The data show that social capital decreased among clients during the pandemic. This has increased problematic substance use, and thus led to a need for assistance. However, in some cases, the existing social capital, such as being in employment, appeared to protect the individual against the acerbation of the SUD. In the following extract clients describe their experiences of the COVID-19 restrictions before entering treatment.

*“In my case, using substances clearly increased. It was among other things, for example, that my best friend started a relationship, and they got stuck at home together. I felt like being totally left alone. And then I just started to hang out in apartments where substances were used—so that somehow, I feel that this pandemic has had a major impact, because there wasn’t anything else to do except to use stuff.”*

(W1 in I2)

*“I have a job that I was not able to do remotely. If I would have had a remote work, it would’ve increased my alcohol use for sure.”*

(W2 in I2)

*“When all places are being closed and there’s nothing to do, and I also have this online addiction, I can’t do anything but sit at my computer and surf online. In my case, being online is closely related to alcohol as well. I sit at my computer and sip wine. I feel nervous about my future after this treatment period, like what am I going to do after this, when there’s nothing to do?”*

(W3 in I2)

*“And then I’ve been thinking about cutting out user friends and other people who use substances from Instagram and Facebook and other platforms, so that I can’t see them fooling around and doing stuff. To make that kind of qualifier.”*

(W1 in I2)

As the quotations reveal, the COVID-19 restrictions seem to have affected the clients’ expectations before treatment in terms of loneliness, boredom, and coping in general. The restrictions have made it more difficult to replace harmful social networks with supportive social networks. Giving up harmful networks can be difficult if the individual is not able to create new ones. The COVID-19 restrictions exposed clients to things that may have made their life situations more difficult before seeking help.

In addition to increased use of substances, the COVID-19 restrictions led to the use of new substances not used before. Because the restrictions hampered drug trafficking, many users used the substances currently available. Below, the clients discuss the effects of the COVID-19 restrictions on the availability of illicit drugs:

*“The COVID-19 restrictions affected the accessibility of illicit drugs, for sure. At least [in the interviewee’s hometown] the amphetamine and Subutex prices went up. Drug trafficking became more difficult due to the movement restrictions, and so on.”*

(M3 in I1)

*“Well, I happen to have pretty exact information about the drug importation. The Subutex prices increased [in one particular city] vastly, and multiple drug loads were intercepted. But the availability of amphetamine, which I personally use, remained the same, as well as cocaine. It didn’t change at all. The availability of Subutex was the only one that changed.”*

(M1 in I1)

*“I noticed the impacts of the COVID-19 restrictions in the Subutex prices as well. The availability of amphetamine remained the same as before.”*

(M2 in I1)

The conversation reveals that the COVID-19 restrictions affected the availability and consequently the price of various illicit drugs. However, according to the interviews, it seems that the effects of the restrictions were local and concerned only certain substances, while the price and availability of other drugs remained the same.

### 4.2. Social Capital Management and Informal Procedural Support

The interviews suggest that the clients’ daily lives were more structured during treatment and included some social support, although this changed due to the COVID-19 restrictions. The clients still became worried about the contrast between the social conditions in inpatient SUD treatment and at home. Due to the COVID-19 restrictions, a client’s social conditions may change radically when they return home. Since their social life is restricted, the contrast between social conditions at home and in treatment may become significantly high.

The COVID-19 restrictions precluded all external visitors from the treatment’s group activities, such as NA groups (*Narcotics Anonymous*). Hence, it appears that the clients were lacking peer support from more experienced peers. According to the data, it seems that the clients did not find the peer support received from other inpatient SUD treatment clients sufficient. The following two quotations describe the clients’ thoughts about peer support:

*“People don’t have experience of being clean. Here, in the treatment unit, you can see that people have experienced only short periods of being drug free, which I don’t consider encouraging, people just inflict their illbeing on others, and I’ve noticed that it spreads easily in these groups here. I consider groups outside the treatment unit very rewarding, but there aren’t groups like that here, because people don’t have experience of being clean for a long time here.”*

(M1 in I6)

*“Personally, I think the same way. In the treatment unit, people don’t have experience of being clean for a longer period of time. It doesn’t generate hope the same way.”*

(W1 in I6)

The duration of the inpatient treatment varied among the clients. However, according to the interviews, all of them seemed to be in the phase of primary desistance. The clients need specific procedural support in their transition from primary desistance to secondary desistance, the phase of maintaining the change. The COVID-19 restrictions undermined the sense of informal procedural support among the clients.

Lack of informal procedural support led to challenges in accumulating new social capital, maintaining existing social capital, and embracing a new way of life. Spending time outside the treatment unit, visits from significant others, along with opportunities for leisure activities, were all restricted due to the pandemic. All of these are normally supportive elements encouraging the clients’ desistance processes during treatment. In the following quotation a client talks about how the restrictions affected the treatment environment:

*“I’m pretty social by nature and I like to have conversations with people, so when there are restrictions on the group activities and leisure activities, so…this is probably one factor that personally disrupts my recovery.”*

(M1 in I5)

*“Yeah, everything like that, I agree with my fellow interviewee. Additionally, of course going out or fetching groceries, for example, it would be nice if you could do something else than just run into the grocery store and back. All contacts and exercise opportunities are limited. We can go out for a walk, but we only have that same stretch of the road where we can walk back and forth. Then we have split some gym sessions and all, but…”*

(M2 in I5)

*“Yeah, the limited exercise opportunities outside the treatment unit. I was expecting greater opportunities before I came here, because I had read the website and I thought that it’ll be nice to get to try out different activities during my time here. Even though COVID-19 was going on, I still for some reason thought that I’d be able to do different leisure activities, and now they’re all gone.”*

(W1 in I5)

### 4.3. Identity

The discussion below reveals that the participants were currently going through an identity change process. The prevailing identity change process appears in the “othering” language that the participants use during interviews:

*“I suppose that you “normal people”, in quotation marks, have bigger problems in adapting to this pandemic than we addicts. You know, we have kind of always lived epidemic times.”*

(M3 in I3)

*“Yes, and under exceptional circumstances. (Laughs).”*

(M1 in I3)

*“Yeah, exceptional circumstances indeed. We have always been kind of isolated.”*

(M3 in I3)

When speaking about themselves, the participants compare “us” to “others”. They seem to identify themselves as “users”, which supports the previous observation of their being in the phase of primary desistance. Based on the interviews, the restrictive measures that targeted home visits, visits from close-ones, and leisure activities have negatively affected the clients’ opportunities to express their changing identities in their own communities.

According to the clients’ experiences, municipal social workers had not been able to meet their clients or visit treatment units similarly as they were before the pandemic:

*“(The social worker) reads about me from other professionals’ records, I don’t know. It’s todays social work, I guess. (Laughs).”*

(M1 in I4)

*“Do you think that it (social work) would be different without the pandemic and all these restrictions?”*

(A2 in I4)

*“Yes, definitely. I’m sure that then they (social workers) would come and visit and keep in touch with me more often, but, personally, I don’t have the energy to talk with them, because I don’t know them either (laughs).”*

(M1 in I4)

Participants felt that in remotely conducted social work their own identity was defined for them through the professional’s interpretation of the client’s former records. However, clients seemed to understand that this was due to the restrictions, and they believed that the social workers would be more in touch with them and become more familiar if there was no pandemic.

### 4.4. Technology and Interaction

Face-to-face interaction was restricted during the pandemic and on some occasions were implemented with technology assistance. Here, two participants talk about their observations about how the restrictions and use of technology have affected interaction in treatment:

*“At Teams-meetings with the community, the connections are tenuous, and sometimes I can’t hear what people are saying. So, it becomes stultifying and boring, as I compare it to what it would be if I could see everyone’s faces. Because I like to observe people and I read people’s gestures and tones. Now, all I can hear is the voice of the professional.”*

(F3 in I2)

*“And then I have to say, that the use of face masks hinders expression of gestures and other non-verbal interaction. I consider it a major restriction that you can’t even see your closest counselor’s gestures or faces when you go through tasks that are important to you. It is a major impact. You know, they are, however, our counselors. They should be able to belong to the community, but now they remain even more distant…”*

(F2 in I2)

According to the interviews, the clients considered the personnel of the treatment unit to be part of the community, but the COVID-19 restrictions largely separated the personnel and clients from each other. Social distancing and using face masks separated these two groups and highlighted hierarchical structures in the client-employee relationships. Clients seem to feel that remote connections make interaction more planned and premeditated, which loses the elements of spontaneity, naturalness, and interactivity.

### 4.5. Inter-Municipal Segregation

According to the data, the clients appeared to be in a decidedly unequal position at the end of the inpatient SUD treatment depending on their existing social networks and their quality. The COVID-19 restrictions accentuated this inequality since they inhibited the formation of new supportive social networks during and after treatment. The clients seemed to have fewer concerns about returning home when they had had stable and supportive social networks, and meaningful activities in their lives before entering treatment. Below, the clients discuss the effects of the restrictions on group activities and peer support opportunities in their own home municipalities:

*“…one of the (NA) groups has totally been shut down, because it used to be held in the city facilities, and the city don’t allow face-to-face meetings at the moment. Then there is another group, where there are fewer meetings as it is, but it seems to be open at least for now. The number of active groups has diminished by half.”*

(M1 in I3)

*“Yes, there is still one active (NA) group (in the interviewee’s hometown). Meetings are held once a week. Yes, face masks are mandatory, but still.”*

(M2 in I3)

*“There are active (NA) groups (in the interviewee’s hometown) every day, but unfortunately the COVID-19 has shut down the best ones, I must admit.”*

(M3 in I3)

According to the data, regional restrictions increased inter-municipal segregation during the pandemic. The relatedness experienced during inpatient treatment enhanced the clients’ awareness of peers’ experiences as well as differences in services and practices between different municipalities.

The clients’ treatment plans after the inpatient treatment depended on their own home municipalities and on the regional COVID-19 restrictions. The participants reported that in smaller municipalities many recovery-related group activities were closed down and the clients’ opportunities to participate in meetings or groups either remotely or face-to-face were variable. Differences in the provision of services puts clients in unequal positions in the service system.

## 5. Discussion

This study investigated how the clients in inpatient SUD treatment felt about the COVID-19 restrictions and their effects on recovery. The results showed that in many cases COVID-19 contributed to an exacerbation of the SUD and life situation before entering treatment. This finding is in line with those of a study by Marsden’s and colleagues’ [8], which suggested that the pandemic may have increased health-related and social risks. During inpatient treatment, due to the restrictions, the clients experienced inadequacy of peer support and informal procedural support. Besides, the function of social capital seemed to have changed and its management became more difficult.

During the interviews, participants tended to use “othering” language when they talked about themselves. By using this discourse, they stressed the differences between “us” and “others”. From the perspective of desistance theories this represents balancing between the current identity and the future possible self. Using othering or dismissive discourse indicates the crystallization of discontent, which nourishes motivation for change in desistance process [20].

This study suggests that managing an individual’s own social capital became more difficult due to the COVID-19 restrictions. Given this perception, clients in treatment during the pandemic may need more support for relinquishing harmful social networks, as well as maintaining and creating new, supportive social networks. Social capital has been identified as fundamental in recovery capital [27,28]. The conception of recovery capital clarifies the role of different resources behind successful recovery.

During the pandemic, inpatient treatment clients appeared to be in a decidedly unequal position vis-à-vis each other when returning home, depending on their existing social networks and the quality of these. Weston and colleagues [28] have stated earlier that, besides the presence or absence of social networks, the nature of those networks should also be taken into consideration when it comes to the successful deployment of social capital. The results of this study corroborate their perceptions.

Social conditions play an important role when it comes to desistance from drugs and/or alcohol. This study suggests that changes in an individual’s social circumstances may not only hamper the desistance process, but also effectively encourage the process. The clients’ social circumstances should be taken into consideration throughout the recovery process. Particular attention should be paid to the differences in social circumstances between treatment and home, especially during the pandemic. According to this study, it would be important to keep these disparities to a minimum.

In this study, the clients felt that the restrictions inhibited interaction between them and the treatment unit personnel. Furthermore, the restrictions seemed to cause dissatisfaction regarding remote group meetings. This observation is in line with Sugarman’s and colleagues’ [38] research, where they found that individuals experiencing SUD were not as satisfied with remote group therapy instead of remote individual therapy, because they were not able to connect as well with other participants as they were in face-to-face meetings. In our study we found that, according to the clients’ experiences, remote connections made interaction more premeditated, which eliminated the elements of spontaneity, naturalness, and interactivity.

Inter-municipal segregation in Finland in organizing SUD services was known to already be a problem before the pandemic [39,40]. In light of these results, this seems to have increased even further under exceptional circumstances. Given this perception, differences in service delivery need to be studied especially during the pandemic, as the execution of the regional evaluation of needs is more demanding under the restrictions. Realistic evaluation of these needs is important since earlier research has shown that most of those in need of substance abuse services had not commenced treatment before the pandemic [40]. A proper regional dimensioning of substance abuse services is a prerequisite for their better accessibility.

Von Greiff’s and Skogens’ [21] study showed that individually tailored assistance becomes significant specifically in the second phase of recovery, which can be compared to the phase of secondary desistance from substances. Thus, it is natural that clients have experienced differences in service delivery. However, in organizing services care should be taken to ensure that all those in need have access to services that meet their subjective requirements.

During inpatient SUD treatment, professionals’ endeavor to help clients to achieve improvements in various life domains, and their interactive approaches have been found to have a major impact on strengthening the client’s agency in everyday life [6,12,29,34]. This study supports these observations while showing that remotely implemented social work has not reached clients the same way as in face-to-face meetings.

Like other studies, this study has its limitations. A common challenge in the field of substance use, and addiction research, is related to the availability of participants. It is also important to note that this study was conducted with a relatively small number of clients in two inpatient SUD treatment units in Finland. Besides, it is noteworthy that more motivated and capable clients are frequently over-represented [41]. The remote implementation of focus group interviews may have affected data collection. The interactive and dialogic characteristics were minimal in these interviews since the participants were obliged to answer the questions each in turn, which was not conductive to joint discussion. However, each focus group interview produced rich data, where all participants were present and participated actively in the discussion.

This information can be utilized in evaluating the impacts of the COVID-19 restrictions and developing new treatment practices under exceptional circumstances. The COVID-19 pandemic offers unique opportunities to develop services and treatment practices for people experiencing SUD to meet the current needs and expectations of post-modern societies [7]. The theoretical background offers an appropriate model for assessing the significance of social capital in desistance from drugs and/or alcohol. Desistance theories deserve more academic attention in the field of addiction research. Implementation of these theories in social work research seems promising. Social workers in various fields meet clients with SUD, hence there is an extensive need for new tools and approaches to these issues. However, the field of substance use services is wide and multi-professional and these results may offer new perspectives and useful information for other practitioners working with people experiencing SUD. This study provides important information about the effect of the COVID-19 restrictions on the recovery and social life of people with SUD.

## 6. Conclusions

This study addressed the influence of the COVID-19 restrictions on inpatient SUD treatment and clients’ experiences of their recovery processes under exceptional circumstances. The results show that the restrictions affected the clients’ recovery processes in multiple ways by exacerbating the severity of the SUD before seeking help, thereby highlighting the need for peer support from more experienced peers, raising concerns about returning home, hindering the expression of a changing identity, as well as intensifying inter-municipal disparities in service delivery. Besides, the pandemic seems to have changed the function of social capital in individual recovery processes. In conclusion, this study suggests that particular attention should be paid to informal procedural support, social capital management, and changing social conditions, among clients who have been in inpatient treatment under pandemic conditions.

Even if social work faced remarkable challenges during the pandemic, these exceptional circumstances also became a significant force for change and advanced innovations in the field [5,7]. However, in social work it is always crucial to consult the target groups about their needs when seeking innovations.

## Figures and Tables

**Table 1:** Interviews and participants.

Number of Focus Group Interviews	
In total	6
Number of participants	
Women	9
Men	10
In total	19
Age of the participants (in years)	
Youngest	23
Oldest	45
On average	33.5

**Table 2:** Original data information.

Transcribed data (in pages)	
In total	60.5
Individual transcribed interview (in pages)	
On average	10.5
The duration of the interview (in minutes)	
Range	43–83
On average	65.8

## Data Availability

Data may be made available upon reasonable request from Katja Kuusisto.
